# The History and Capabilities of Herbal Simples

**Published:** 1891-04-25

**Authors:** W. T. Fernie


### The History and Capabilities of Herbal Simples.

By W. T. Fernie, M.D.

*(Continued from page 370, Vol. IX.)*

### XXX.—THE DOCK.

Even from the time of Josephus, the historian, children have been wont in their games to select their playmates by means of the rhyme, "Hickory, dickory, dock; the mouse ran up the clock; the clock struck one; the mouse was gone; O-U-T spells 'out.'" I take it that hickory and dickory were merely fanciful words leading up to dock, which was the name given in early days to a wide-spread tribe of broad-leaved wayside weeds. The term meant originally a bundle of hemp, and corresponded to a similar word signifying a flock. It was first borne by the bur dock (*Arctium lappa*), from the frequent entanglement of its seed vessels by their small hooked spines in the wool of sheep passing along the hedgerows. Then the name got to include other broad-leaved herbs, all of the sorrel kind, and used in pottage, or medicine. They belong to the botanical order of *polygonaceæ*, or many-kneed; because, like the wife of Yankee-Doodle, famous in song, they are "double-jointed," though he, poor man, thinking to find Mistress Doodle doubly active in her household duties, was, as the rhyme says, "sadly disappointed."

Nowadays there are several sort of dock, some of which are cultivated, such as the monks' rhubarb, or herb patience, an excellent pot herb; whilst others grow wild in meadows and by river sides, such as the round-leafed dock, the sharp-pointed dock, the sour dock, and the great water-dock. All these resemble our garden rhubarb more or less, in their general characteristics, and in possessing much tannin. Most of them chemically furnish rumicine, or crysophanic acid, which is highly useful in several chronic diseases of the skin among scrofulous patients. A generic name applied to some of the docks is *rumex*, from the Hebrew, *ramach*, a spear; others are called *lapathum*, from the Greek *lapazein*, to cleanse, because they act medicinally as purgatives. The leaves of the common wayside dock are often applied as a rustic remedy to burns and scalds, also to dress blisters. Likewise a popular cure for nettle stings is to rub them with a dock leaf, whilst saying, "Out nettle! In dock! Dock shall have a new smock!" or "Nettle out; dock in; Dock remove the nettle sting!" Chaucer alludes to this bit of folk lore in "Troilus and Cressida;" but is supposed rather to mean the common round-leaved mallow, which is still favoured by children for the same purpose. Dock tea made from the root of the common broad-leaved dock was formerly given for the cure of boils. This plant is often called butter-dock, because its leaves are used for wrapping up butter. It is the most common of all the docks, being large and spreading, and so coarse that cattle refuse to eat it.

The yellow curled dock (*rumex crispus*), so-called because its leaves are crisped at their edges, grows freely in our roadside ditches and waste places. A tincture is made for medicinal purposes from this plant before it flowers. It proves curative for a dry tickling cough, which seems to come from the windpipe, violent and fatiguing in its character, whilst with but little expectoration. It is likewise of use for any obstinate itching of the skin, in which respect it was singularly beneficial for the contagious army itch which prevailed during the late American War. To be applied externally an ointment may be made by boiling the root in vinegar until its fibre is softened, and by then mixing the pulp with lard. From five to ten drops of the tincture may be given with a tablespoonful of cold water, two or three times a day.

The great water dock (*rumex hydrolapathum*) is the celebrated Herba Britannica of Pliny, so-called not because it is of British origin, but from three Teuton words: *brit*, to tighten; *tan*, a tooth; and *ica*, loose; thus expressing its power of fastening loose teeth, or of curing the diseased condition which makes them become loose. The root of this herb when finely powdered is commonly employed as a dentifrice for the above purpose by Swedish ladies, and is also given in scurvy. Gargles prepared from this root are excellent for sore throat and for relaxed uvula. All the docks "being boiled do soften and mollify the belly." Horace recognises this fact (Sermonum Satyr, 4):—

"Si dura morabitur alvus,
Mytulus et vites pellent obstantia conchæ,
Et Lapathi brevis herba."

The herb patience, or monks' rhubarb—a Griselda among plants—may be similarly given with admirable effect in pottage as a domestic aperient, "loosening the belly, helping the jaundice, and dispersing the tympany."

Our garden rhubarb, which is also a true dock, and which we take in puddings or tarts, contains a free quantity of oxalic acid, and is therefore objectionable as food for gouty persons liable to derangements of the kidneys, especially if at the same time hard water is drunk, which contains lime. Oxalate of lime will then be formed, and will probably bring on a sharp attack of renal gout.

Sorrel, or "sour" dock, is equally objectionable in the same cases for a like reason, though the leaves of this plant form the chief constituent of the "soupe aux herbes" which a French lady will order for herself after a long and tiring journey. Sorrel bears further the names of soursabs, sourgrabs, soursuds, soursauce, and greensauce. Because of their acidity the leaves make a capital sauce for stewed lamb, veal, or sweetbread. In Ireland they are eaten with fish and with other alkalescent food. For a similar reason, as corrective of scrofulous deposits, sorrel is chiefly beneficial in the general cure of scurvy. This and the angelica are the only plants used by the Laplanders as food, except berries. Applied externally the bruised leaves will purify foul ulcers. Says John Evelyn, in his noted "Acetaria" (1729): "Sorrel sharpens the appetite, assuages heat, cools the liver, and strengthens the heart; it is an antiscorbutic, resisting putrefaction, and in the making of salads imparts so grateful a quickness to the rest as supplies the want of oranges and lemons. Together with salt, it gives both the name and the relish to sallets, from the sapidity which renders not plants and herbs only, but men themselves and their conversations pleasant and agreeable. But of this enough, and perhaps too much, lest while I write of salts and sallets, I appear myself *insipid.*"

